# The Effects of Octapeptin Supplementation on Growth Performance, Serum Biochemistry, Serum Immunity, and Gut Microbiota in Weaned Piglets

**DOI:** 10.3390/ani14172546

**Published:** 2024-09-02

**Authors:** Sheng Huang, Li Yang, Li Wang, Yu Chen, Xiuliang Ding, Feiyun Yang, Shiyan Qiao, Jinxiu Huang

**Affiliations:** 1 Chongqing Academy of Animal Sciences, Chongqing 402460, China; huangs@cqaa.cn (S.H.); sddingxl@aliyun.com (X.D.); yfeiyun@yeah.net (F.Y.); 2 National Center of Technology Innovation for Pigs, Chongqing 402460, China; lekalter@163.com (L.Y.); q15928806587@163.com (L.W.); 15222269277@126.com (Y.C.); 3 State Key Laboratory of Animal Nutrition and Feeding, Ministry of Agriculture and Rural Affairs Feed Industry Centre, China Agricultural University, Beijing 100193, China; qiaoshiyan@cau.edu.cn

**Keywords:** weaned piglets, octapeptin, growth performance, fecal microbiota, feed additives

## Abstract

**Simple Summary:**

Weaning is a critical and challenging period for piglets, often leading to stress, poor growth, and increased disease incidence. This study investigated using octapeptin as a feed additive for weaned piglets. The results indicated that octapeptin significantly improved diarrhea and enhanced feed conversion ratio by modulating immunity and reducing inflammation compared to standard diets. Octapeptin also decreased TNF-α levels, boosted the immune system, and increased beneficial bacteria, such as *Collinsella* and *Olsenella*, positively impacted gut health. These findings suggested that octapeptin is a promising, safe, natural antibiotic alternative that promotes health and growth in weaned piglets.

**Abstract:**

With the prohibition of antibiotics in animal feed, the livestock industry faces significant challenges, including increased morbidity and mortality rates and reduced farming efficiency. Developing green, natural, and safe antibiotic alternatives has become a research hotspot. This study evaluated the effects of octapeptin as a feed additive on growth performance, diarrhea incidence, serum biochemistry, serum immune factors, and gut microbiota of weaned piglets. Seventy-two weaned piglets were randomly assigned to three groups based on body weight and sex, with each group receiving different dietary treatments: a negative control group (CON, basal diet), a positive control group (MC, basal diet + 5 mg/kg Microcin C7), and an octapeptin supplement group (OP, basal diet + 40 mg/kg octapeptin). After 28 days of feeding experimental diets, the results demonstrated that supplementing the diet of weaned piglets with octapeptin significantly improved the feed conversion ratio compared to the control group (*p* < 0.05) over the entire experimental period. Furthermore, a reduction in diarrhea incidence was observed during the late nursery period (14–28 d), resulting in an overall improvement in diarrhea compared to the other two groups (*p* < 0.01). Serum biochemical analysis results revealed a trend towards decreased alanine aminotransferase level in the octapeptin group, with no significant differences in other indicators, suggesting potential improvements in liver function without causing liver damage. In addition, compared to the control group, octapeptin enhanced mucosal immunity by decreasing TNF-α level (*p* < 0.05). Fecal microbiota analysis results showed a significant increase in beneficial bacteria such as *Collinsella* and *Olsenella* in the octapeptin group compared to the other two groups (*p* < 0.05), indicating a positive impact on gut health. These findings supported the potential of octapeptin as an alternative to antibiotic growth promoters in weaned piglets’ diets.

## 1. Introduction

Since the 1950s, following Moore’s report on the benefits of antibiotics in promoting growth and enhancing disease resistance in broiler chickens [1], the use of antibiotics in animal husbandry has surged [2,3]. This increase has led to antibiotic residues in livestock [4], food safety concerns [5], and environmental pollution [6,7], posing potential threats to both ecosystems and human health [8]. Controlling antibiotic resistance and reducing antibiotic use have become national priorities and research hotspots in many countries. Since 1986, several countries, including Sweden, the European Union, the United States, Japan, and South Korea, have banned antibiotics in animal feed for growth promotion [9,10]. China implemented a comprehensive ban on growth-promoting antibiotics in July 2020 [11]. Consequently, identifying and developing alternative agents that can promote growth, alleviate diarrhea, and maintain intestinal health has become urgent.

Antimicrobial peptides (AMPs) exhibit antibacterial [12,13,14,15], antiviral, and immunomodulatory activities, with low toxicity and minimal side effects [16] and no bacterial resistance [17,18,19]. These peptides are derived from diverse sources, including animals, plants (such as buferin, beta-defensin 2, AMP-A3, cecropin), bacteria (such as colicin and octapeptin), and fungi (such as plectasin and peptaibols), positioning them as promising candidates for alternative antimicrobial therapies [20]. They have broad potential applications in disease treatment [21], food preservation [22,23], and as feed additives [24,25,26]. In livestock production, studies on piglets have shown that AMPs like colicin, cecropin, and defensin have significant growth-promoting effects, increasing final weight, average daily gain, and feed conversion rate while reducing diarrhea incidence [27]. AMPs also elevate glutathione levels, total antioxidant capacity, and peroxidase activity in serum and intestines, significantly increasing IgA and IgG levels while decreasing TNF-α, IL-1β, and IL-6 [28,29]. Further research has shown that AMPs increase beneficial bacteria like *lactobacilli* and *bifidobacteria* and reduce *E. coli* in the gut [26,30,31]. AMPs positively impact intestinal villus height, crypt depth, and gut barrier function [32,33]. As a dietary supplement, AMPs can enhance the humoral immunity of piglets [34], increasing the levels of immunoproteins such as IgG, IgM, and IgA. Additionally, AMPs can improve cellular immunity by increasing the number and proliferative function of peripheral T-cell subsets in piglets [35].

Due to the accumulation of antibiotic-resistant genes, there is an urgent need for alternatives to antibiotics that can effectively combat resistant bacterial strains without promoting further resistance. Octapeptin, a broad-spectrum lipopeptide produced by *Bacillus circulans*, has been reported to meet these requirements [36]. Research has demonstrated that octapeptin exhibits activity against polymyxin-resistant bacteria [37]. Meanwhile, octapeptin is less likely to induce resistance and does not exhibit cross-resistance with polymyxins [37], positioning it as a promising candidate for combating resistant bacterial strains. Currently, the in vitro and in vivo activity of octapeptin has been evaluated through antibacterial assays and mouse models [37]. In vitro studies have found that octapeptin has a MIC of about 2~8 µg/mL against some Gram-negative bacteria, which is 8–32 times lower than polymyxin B [38]. In vivo studies found octapeptin to have lower nephrotoxicity and a longer half-life than polymyxin B [39].

Weaning is one of the most challenging stages in pig production, significantly impacting growth performance [40,41]. It induces stress responses in pigs, disrupts intestinal digestion and absorption capabilities, compromises gut health [42], markedly reduces growth and immunity in weaned piglets [43,44], and increases the incidence of diarrhea [45]. Therefore, mitigating weaning stress may result in improved performance, and octapeptin may have beneficial effects when used at appropriate levels. Although numerous studies have demonstrated that antimicrobial peptides possess both antimicrobial properties and immunomodulatory effects [46,47,48], research specifically focusing on the immunomodulatory effects of octapeptin is almost non-existent. Therefore, this study aims to evaluate the effects of octapeptin as a feed additive on growth performance, diarrhea incidence, serum biochemical indices, serum immune factors, and intestinal microbiota in weaned piglets, with the expectation of preliminarily exploring the immunomodulatory activity of octapeptin.

## 2. Materials and Methods

All procedures of this experiment were approved by the Institutional Animal Care and Use Committee of Chongqing Academy of Animal Sciences (Chongqing, China). The experiment was conducted at the Shuanghe Research Base of the Chongqing Academy of Animal Sciences.

### 2.1. Preparation of Antimicrobial Octapeptin

Our laboratory provided the antimicrobial octapeptin sample (Chongqing, China), which had a purity of 95%, and used corn flour as the carrier. The octapeptin was derived from *Bacillus circulans*.

### 2.2. Animals, Diets, and Experimental Design

Seventy-two 28-day-old weaned piglets (Duroc × Landrace × Yorkshire), with an initial body weight of 6.57 ± 0.08 kg, were used in this study, which lasted for 28 days. Upon arrival at the experimental facility, littermates were randomly assigned to different groups to avoid confounding effects. The piglets underwent a 7-day feed adaptation period, after which they were assigned to three groups using a randomized complete block design, with sex and body weight at weaning as blocking factors. Each treatment group consisted of 6 replicates, with each replicate containing 4 piglets (2 males and 2 females). The dietary treatments were as follows: a negative control group (CON, basal diet), a positive control group (MC, basal diet + 5 mg/kg Microcin C7), and an OP treatment group (OP, basal diet + 40 mg/kg octapeptin). Microcin C7, used in the positive control group at a dosage of 5 mg/kg (purity 95%), is a narrow-spectrum antimicrobial peptide composed of seven amino acids, known for its low cross-resistance [49], making it suitable as a feed additive. Research has demonstrated that an addition of 500 mg/kg (with a purity of 1.19%) of Microcin C7 has beneficial effects on growth performance, diarrhea incidence, apparent total tract digestibility (ATTD) of ether extract and Ca, immune performance, intestinal morphology, and microbiota structure in piglets [28]. All diets were formulated to meet the nutrient requirements recommended by NRC (2012), and the ingredient composition and nutrient content of the basal diet are presented in Table 1.

### 2.3. Animal Management

Seventy-two piglets were kept in the experimental pens with plastic slatted floors, with 4 piglets per pen (1.4 m × 2 m). Each pen was fitted with an adjustable stainless-steel feeder and a duckbill drinker to allow piglets to eat and drink ad libitum. The pig housing environment was meticulously controlled, including regulation of CO_2_ and ammonium levels in the air, ventilation rates, humidity, and temperature. The average indoor temperature was maintained between 24 and 26 °C, while relative humidity was kept at 60–70%. To mitigate disease risks, the experimental facilities underwent daily cleaning, supplemented by weekly health assessments of the piglets conducted by a veterinarian. In this study, piglets would undergo a 12 h fasting period before weight measurements on days 0, 14, and 28, alongside weighing the remaining feed in each pen. These data points were used to calculate the average daily gain (ADG), average daily feed intake (ADFI), and feed-to-gain ratio (F/G). The diarrhea index (DI) and diarrhea rate (DR) were assessed using the formula diarrhea index = sum of diarrhea scores/(total number of piglets × number of days tested), diarrhea rate (%) = the total number of diarrhea piglets/(total number of piglets × number of days tested) × 100%. A higher diarrhea rate indicated more severe diarrhea among the piglets. Fecal consistency was assessed and scored based on the following criteria [50]: normal feces, characterized by formed or granular stools, were assigned a score of 0; mild diarrhea, indicated by soft but formed stools, was given a score of 1; moderate diarrhea, identified by thick, unformed stools without separation of fecal water, received a score of 2; and severe diarrhea, marked by watery, unformed stools with separation of fecal water, was scored as 3. Any score above 0 indicated the presence of diarrhea and was included in the calculation of the diarrhea rate.

### 2.4. Sample Collection

On the morning of day 28, two piglets (one male and one female) with the average body weight from each replicate group were selected for blood sampling. Blood was collected from the jugular vein of fasted piglets using a 10 mL syringe and placed into pro-coagulant blood collection tubes. The samples were left to stand for 2–3 h and then centrifuged at 1000× *g* for 10 min at 4 °C. The supernatant was aliquoted into four 1.5 mL centrifuge tubes and stored at −20 °C for future analysis.

On day 28th, fresh fecal samples were collected from pigs on a per-pen basis. Using a sterile spoon, the central portion of the feces, which had not come into contact with the ground, was extracted and placed into sterile zip-lock bags. After collecting half a bag of feces, the samples were thoroughly mixed, resulting in 30 fecal samples. These samples were then transported to the laboratory and stored at −80 °C for subsequent 16S rRNA high-throughput sequencing to analyze the fecal microbiota.

### 2.5. Serum Biochemical and Immune Parameters

An automated biochemical analyzer was used to measure the levels of total bile acid (TBA), total protein (TP), albumin (ALB), globulin (GLB), total bilirubin (TBil), blood urea nitrogen (BUN), cholesterol (CHO), and triglycerides (TG) in serum. The activities of alanine aminotransferase (ALT), aspartate aminotransferase (AST), and gamma-glutamyl transferase (GGT) were also assessed. All procedures followed the manufacturer’s instructions for the automatic biochemical analyzer (Beckman Coulter AU5800; Brea, CA, USA).

Serum concentrations of immunological factors, including IgG, IgM, IgA, IL-2, IL-6, IL-10, and TNF-α, were determined using ELISA kits according to the manufacturer’s instructions. All cytokine ELISA kits were obtained from Thermo Fisher Scientific (Waltham, MA, USA).

### 2.6. Microbiota Composition by 16S rRNA Sequencing Analysis

The DNA extracted from fecal genomic samples was checked using 1% agarose gel electrophoresis. Sequencing adapters were added to the ends of primers 338F and 806R, and the V3-V4 gene fragments were PCR-amplified. The PCR products were purified, quantified, and normalized to construct a paired-end (PE) library. After quality control, the library was sequenced using an Illumina MiSeq platform.

The paired-end reads obtained from MiSeq sequencing were split according to samples and underwent quality control and filtering based on sequencing quality. The paired-end reads merged based on their overlap to generate optimized sequences. These optimized sequences were then processed using sequence denoising methods (such as DADA2 or Deblur) to obtain amplicon sequence variant (ASV) representative sequences and abundance information. Based on the ASV representative sequences and abundance data, alpha diversity analyses were conducted on the Majorbio Cloud Platform (https://cloud.majorbio.com/, accessed on 29 June 2024) to assess species diversity within individual samples. Indices such as Chao, Shannon, Simpson, and Coverage were calculated at a 97% similarity level, and rarefaction curves were plotted. Beta diversity analyses were performed to compare community composition and structural differences among different groups, with principal coordinate analysis (PCoA) plots generated based on distance matrices.

### 2.7. Statistical Analyses

Data on piglet growth performance, blood biochemistry, and serum immunity were analyzed using the GLM procedure in SAS software (version 9.2, SAS Institute Inc., Cary, NC, USA) through analysis of variance (ANOVA) followed by Tukey multiple range tests, with *n* = 24 replicates per treatment group. The experimental unit for growth performance measurements was the pen, while for serum immunity and blood biochemistry, the experimental unit was the serum sample collected from each piglet. The chi-square test was used to analyze the diarrhea index and rate, with each piglet serving as the experimental unit. The Wilcoxon rank-sum test compared the relative abundance of microbial communities, considering each piglet as an independent experimental unit. Differences were considered statistically significant at *p* < 0.05 and were regarded as trends when 0.05 < *p* ≤ 0.1. Superscripts were added in each table to indicate tendencies where applicable.

## 3. Results

### 3.1. Effect of Octapeptin on the Growth Performance of Piglets

The growth performance of piglets’ results, shown in Table 2, proved that adding octapeptin to the diet of weaned piglets significantly reduced the F/G throughout the entire experimental period (*p* < 0.05). There was no effect on ADG and body weight. During the entire experimental period, the ADFI of the MC group was significantly reduced by 10.82% compared to the CON group (*p* < 0.05), with a tendency of reduction observed in the 14–28 d (*p* < 0.1). The ADFI of the OP group was intermediate between the MC and CON groups, with no significant difference observed among the groups. According to Table 3, the OP group exhibited a remarkable reduction in diarrhea index and diarrhea rate compared to the CON and MC groups for days 14 to 28 (*p* < 0.01), resulting in a decrease in diarrhea index and diarrhea rate over the entire experimental period (*p* < 0.05).

### 3.2. Effect of Octapeptin on Serum Biochemical Indexes of Piglets

The effects of octapeptin supplementation on the serum biochemical parameters of weaned piglets are detailed in Table 4. Compared to the CON group, ALT levels in the MC group were markedly reduced by 41.52% (*p* < 0.05). The OP group showed ALT levels that were intermediate in value between the CON and MC groups. No significant changes were observed in other biochemical parameters. Additionally, a decrease of 13.06% in CHO level was observed in the MC group (*p* < 0.1), while the CHO level in the OP group decreased by 7.90%.

### 3.3. Effect of Octapeptin on Serum Immunity Indexes of Piglets

The effects of adding octapeptin to the diet on the immune factors of weaned piglets are shown in Table 5. The IgG level in the OP group was markedly reduced by 61.77% compared to the MC group (*p* < 0.01), with no significant difference observed relative to the CON group. Meanwhile, the TNF-α level in the OP group was comparable to the MC group but notably decreased by 27.85% compared to the CON group (*p* < 0.05). It was also noted that the IgA level in the MC group increased significantly by 33.33% compared to the CON group (*p* < 0.05), while the IgA level in the OP group showed no difference compared to either the MC or CON group.

### 3.4. Fecal Microbiome Analysis

**Figure 1:**
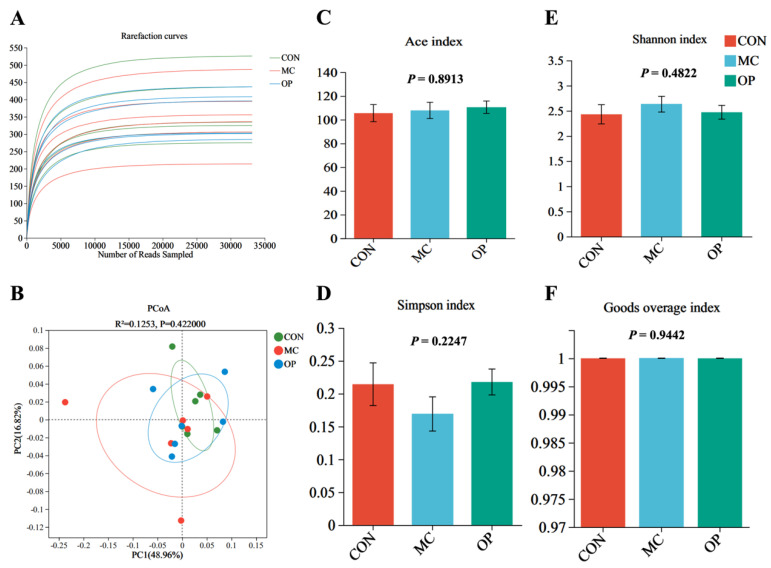
Analysis of fecal microbiome diversity and composition in piglets. Rarefaction curves (**A**). Principal coordinate analysis (PCoA) plot based on Bray–Curtis distances, illustrating the clustering of microbial communities (**B**). Alpha diversity of cecal flora (**C**–**F**). Error bars represent the standard error of the mean. Statistical significance was determined using ANOVA.

The rarefaction curves from the 16S rRNA high-throughput sequencing, shown in Figure 1A, indicated that all treatment groups (CON, MC, OP) reached a plateau, demonstrating that the sample sizes were sufficient to cover most microbial diversity. The PCA plot illustrated the distribution of microbial community structures across different treatment groups, with PC1 and PC2 explaining 48.96% and 16.82% of the variance, respectively (Figure 1B). No significant differences were observed in the Ace index, Simpson index, Shannon index, or Goods coverage index among the CON, MC, and OP groups (Figure 1).

**Figure 2:**
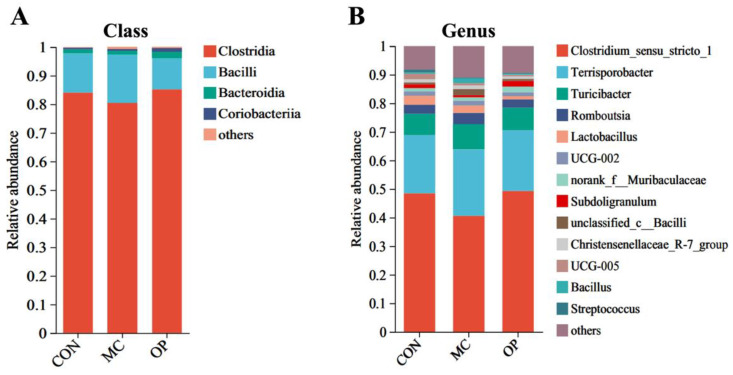
Relative abundance of piglet fecal microbiota at class and genus levels across different treatment groups. *Clostridia*, *Bacilli*, *Bacteroidia*, and *Coriobacteriia* are the dominant classes (**A**). Relative abundance of fecal microbiota at the genus level. The dominant genera include *Clostridium* sensu stricto 1, *Terrisporobacter*, *Turicibacter*, and others (**B**).

**Figure 3:**
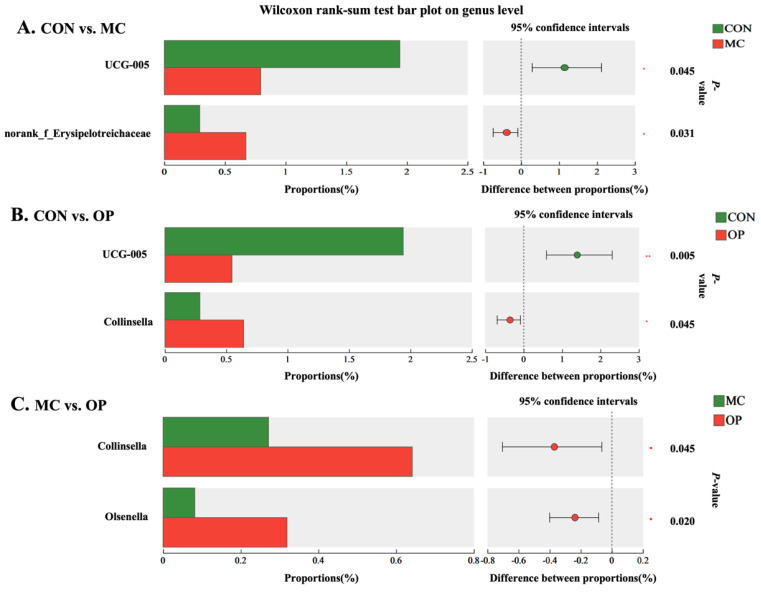
Wilcoxon rank-sum test bar plots of fecal microbiota at the genus level across different treatment groups. Differential genera UCG-005 (*p* = 0.045) and norank_f_*Erysipelotrichaceae* (*p* = 0.031) between CON and MC groups (**A**). Differential genera UCG-005 (*p* = 0.005) and *Collinsella* (*p* = 0.045) between CON and OP groups (**B**). Differential genera *Collinsella* (*p* = 0.045) and *Olsenella* (*p* = 0.020) between MC and OP groups (**C**).

The relative abundances at the class level for all treatment groups were displayed in Figure 2A, highlighting that the top four dominant classes were *Clostridia*, *Bacilli*, *Bacteroidia*, and *Coriobacteriia*. *Clostridia* constituted the most significant portion of the fecal microbiota, with relative abundances exceeding 80%. At the genus level, 45 genera were identified across all samples after data normalization, with the top 13 genera shown in Figure 2B. Statistical analysis using the Wilcoxon rank-sum test compared the relative abundances of microbial communities at the genus level among the three experimental groups, as depicted in Figure 3.

Compared to the CON group, the MC and OP groups substantially reduced the abundance of UCG-005 (*p* < 0.05), while the MC group had a notable increase in the genus norank_f_*Erysipelotrichaceae* (*p* < 0.05), and the OP group exhibited a marked increase in the genus *Collinsella* (*p* < 0.05). Furthermore, the OP group had significantly higher abundances of *Collinsella* and *Olsenella* than the MC group (*p* < 0.05).

## 4. Discussion

Numerous studies have demonstrated that feed additives can enhance intestinal immunity and regulate the gut microbiota in piglets [51,52,53], thereby mitigating the adverse effects of weaning and other environmental challenges [54,55]. Antimicrobial peptides are considered one of the best antibiotic alternatives [56]. In this study, octapeptin, an antimicrobial peptide with bactericidal and immunomodulatory activities, was evaluated for its efficacy and safety as a potential feed additive for weaned piglets. 

This study suggests that octapeptin enhances the feed conversion ratio by mitigating diarrhea. It is worth noting that both the control and treatment groups experienced varying degrees of diarrhea, which is speculated to be caused by environmental changes and dietary shifts following weaning. The first two weeks post-weaning are the most stressful for piglets, as environmental and dietary changes can significantly affect their feed intake and body weight [41]. Research indicates that antimicrobial peptides can alleviate inflammation and reduce the energy expenditure associated with immune responses, which allows more nutrients to be utilized for growth, thus improving the feed conversion ratio [57]. Moreover, the observed reduction in feed intake may be linked to a satiety effect induced by changes in the gut microbiome, which can modulate appetite and feeding behavior [58]. These changes could decrease feed intake without compromising the energy available for growth. Research has shown that the supplementation of Microcin C7 can improve ATTD by increasing the availability of nutrients for intestinal absorption and inducing changes in intestinal morphology, epithelial thickness, and epithelial cell turnover, thereby enhancing feed conversion [59]. This mechanism differs from that of octapeptin, which improves feed conversion primarily by alleviating diarrhea.

Blood biochemical parameters are critical indicators of nutritional levels, endocrine status, and overall health, reflecting changes in tissue cell permeability and metabolism and indicating organ function and condition [60,61]. ALT is an enzyme primarily found in liver cells involved in protein metabolism and accelerates the conversion of amino acids in the body. Even a 1% destruction of liver cells can double serum enzyme levels, making ALT a marker enzyme for acute liver cell damage [62]. The study showed a notable reduction in the ALT level in the Microcin C7 group compared to the control group. The octapeptin group showed ALT levels that were intermediate values between the control and Microcin C7 groups, with no considerable changes in other biochemical parameters, suggesting a possible improvement in liver function without causing hepatic injury.

Cytokines play crucial roles in immune responses and inflammation, mediating susceptibility to infections and gastrointestinal disorders [63]. AMPs have been found to regulate cytokine levels. For example, Yi et al. [64] reported that feed supplemented with Cathelicidin-WA, an AMP derived from the snake *Bungarus fasciatus*, reduced serum levels of the pro-inflammatory cytokine IL-6. In the same vein, the present study observed that octapeptin markedly reduced TNF-α levels. It was also noted that IgA level significantly increased in the Microcin C7 group compared to the control group, with the octapeptin group exhibiting intermediate levels between the two. These findings suggest that octapeptin supplementation may have alleviated inflammation to some extent and helped maintain immune homeostasis in piglets. The immature intestinal immune function of newly weaned piglets at 2–4 weeks increases their disease susceptibility [44,65]. Small fluctuations in immune factors can trigger an imbalance in immune homeostasis, leading to inflammation or diarrhea [66]. Several in vitro and in vivo studies have shown that the concentration of pro-inflammatory cytokines in the small intestine of piglets usually increases after weaning [44]. High levels of IL-6 can damage tissues, while TNF-α disrupts epithelial barrier function [67]. Additionally, weaned piglets’ IgA concentrations remain low until 50 days of age [68]. Intestinal inflammation is often associated with impaired intestinal barrier function, making it easier for pathogens to invade and cause diarrhea [35]. Therefore, this study preliminarily suggests that octapeptin may regulate gut immune homeostasis in weaned piglets by modulating immune factor levels, such as TNF-α. This regulation could enhance the intestinal defense mechanisms, reduce inflammatory responses, and consequently lower the incidence of diarrhea.

Weaning impairs piglets’ intestinal epithelial barrier function, disrupting gut microbial balance [69]. Once this balance is disturbed, potential pathogenic bacteria can invade and colonize the intestine [70]. Weaning stress also causes a sharp reduction in feed intake, limiting nutrients for bacterial survival and proliferation [71]. *Salmonella* and enterotoxigenic *E. coli* can use ethanolamine as a carbon or nitrogen source, gaining a nutritional advantage over other microbiota [72]. Enterotoxigenic *E. coli* also uses fucose to activate the type III secretion system, promoting pathogen adhesion to host intestinal cells [73]. Consequently, weaned piglets are more prone to intestinal inflammation and post-weaning diarrhea due to rapid pathogen proliferation and loss of microbial diversity [74]. Recent studies have reported that dietary supplementation with AMPs, such as lactoferrin and lactoferrin fusion peptides, small peptide-chelated iron, AMP A5 (A3), and cecropin AD, benefits host animals by reducing pathogenic bacteria and increasing beneficial lactic acid bacteria, thereby improving the gut environment in weaned pigs by enhancing gut barrier function [27]. Our fecal microbiota alpha and beta diversity analysis showed no notable differences between groups. However, this study provides preliminary evidence that the addition of octapeptin leads to significant microbial changes, particularly the notable increase in beneficial genera *Olsenella* spp. and *Collinsella.* Based on the findings from previous analyses of growth performance, diarrhea incidence, and immune factors, we can reasonably speculate that the initial mechanism of octapeptin as a feed additive in weaning piglets lies in maintaining immune homeostasis through the regulation of immune factors, which in turn enhances gut defense, promotes the colonization of beneficial bacteria, and collectively contributes to the reinforcement and maintenance of gut health. The increase in the number of *Olsenella* spp. and *Collinsella* was positively correlated with the increase in IL-10, which may help to maintain the diversity and stability of gut microbes and reduce the colonization of harmful bacteria [75,76]. These bacteria are involved in various metabolic activities, including short-chain fatty acid production, which is crucial for gut health and barrier function [77,78]. Given that the mechanisms by which octapeptin regulates the microbiome are not fully understood, this study provides experimental evidence for octapeptin as a potential treatment for post-weaning diarrhea and offers insights for future microbiome research.

## 5. Conclusions

In this study, adding octapeptin to the diets of weaned piglets significantly improved feed conversion ratio and alleviated diarrhea compared with the control group. Octapeptin supplementation has the potential to improve liver function. Additionally, it significantly reduced TNF-α levels, enhancing mucosal immunity. Octapeptin positively influenced the fecal microbiota, considerably increasing the abundance of beneficial bacteria such as *Olsenella* and *Collinsella*. These findings suggest that octapeptin is a promising alternative to traditional antibiotic growth promoters in piglets, offering a natural and safe option for enhancing piglet health and growth performance.

## Figures and Tables

**Table 1:** Ingredient composition and nutrient content of the basal diet (as-fed basis, %).

Ingredient, %	Content	Nutrient Level	Measured
Corn	56.18	Metabolizable Energy, Kcal/kg ^1^	3444.44
Extruded Corn	8.00	Crude Protein	20.6
Soybean Meal, 46%	17.50	Calcium	0.59
Extruded Soybean Flour	4.00	Available Phosphorus	0.34
Fermented Soybean Meal	5.50	Total Lysine	1.34
Yeast Culture	2.00	Total Methionine	0.43
Glucose	1.00	Total Methionine + Cysteine	0.77
Fish Meal	1.00	Total Threonine	0.82
High-Fat Powder	1.00	Total Tryptophan	0.22
Calcium Formate	0.80		
Dicalcium Phosphate	0.60		
Salt	0.35		
Acidifier ^3^	0.80		
Lysine, 98.5%	0.40		
Choline Chloride, 50%	0.10		
DL-Methionine, 99%	0.10		
Threonine, 99%	0.08		
Complex Enzymes	0.07		
Premix ^2^	0.52		
Total	100		

^1^ The metabolizable energy value of the diet was calculated according to the proportion of each ingredient in the formulation and the data in the NRC (2012). ^2^ The premix provided the following nutrients per kilogram of feed: Copper (Cu) 5.12 mg, Iodine (I) 0.15 mg, Iron (Fe) 87.59 mg, Manganese (Mn) 3.69 mg, Selenium (Se) 0.30 mg, Zinc (Zn) 84.43 mg, Vitamin A (VA) 9000 IU, Vitamin D (VD) 3000 IU, Vitamin E (VE) 24 IU, Vitamin K (VK) 3 mg, Thiamine 3 mg, Riboflavin 7.5 mg, Pantothenic Acid 15 mg, Niacin 30 mg, Pyridoxine 3.60 mg, Biotin 0.15 mg, Folic Acid 1.50 mg, and Vitamin B12 (VB12) 0.036 mg. ^3^ Acidifier: The composition includes 35% phosphoric acid, 5% citric acid, 10% fumaric acid, 5% benzoic acid, 5% calcium formate, and 40% SiO_2_.

**Table 2:** The effect of dietary octapeptin supplementation on growth performance in piglets.

Items	CON	MC	OP	SEM	*p*-Value
BW, kg					
0 d	6.62	6.56	6.54	0.08	0.72
14 d	8.97	9.34	9.00	0.18	0.33
28 d	14.79	15.04	15.31	0.46	0.73
ADG, g/d					
0–14 d	173.77	197.96	171.61	9.50	0.13
14–28 d	415.71	407.06	412.48	20.37	0.96
Whole period	291.93	302.51	309.42	16.36	0.75
ADFI, g/d					
0–14 d	269.40	261.37	245.91	11.30	0.35
14–28 d	682.46	590.34	630.26	26.54	0.08
Whole period	475.93 ^a^	424.45 ^b^	433.71 ^ab^	13.64	0.04
F/G					
0–14 d	1.56 ^a^	1.36 ^b^	1.41 ^ab^	0.04	<0.01
14–28 d	1.66	1.46	1.53	0.08	0.19
Whole period	1.65 ^a^	1.40 ^b^	1.43 ^b^	0.07	0.04

CON = basal diet without additive; MC = basal diet + 5 mg/kg Microcin C7; OP = basal diet + 40 mg/kg octapeptin. BW: body weight; ADG: average daily body gain; ADFI: average daily feed intake; F/G: ratio of feed to gain. SEM, standard error of the mean. The following tables were the same as this table. ^a,b^ Means listed in the same row with different superscripts are significantly different (*p* ≤ 0.05).

**Table 3:** The effect of dietary octapeptin supplementation on diarrhea index and diarrhea rate in piglets.

Items	CON	MC	OP	SEM	*p*-Value
DI					
0–14 d	0.52	0.47	0.46	0.10	0.12
14–28 d	0.57 ^a^	0.60 ^a^	0.43 ^b^	0.13	<0.01
Whole period	0.54 ^a^	0.53 ^a^	0.45 ^b^	0.10	0.02
DR, %					
0–14 d	10.71	10.42	9.40	2.99	0.52
14–28 d	11.16 ^a^	12.26 ^a^	6.75 ^b^	3.10	<0.01
Whole period	10.94 ^a^	11.26 ^a^	8.06 ^b^	2.41	<0.01

CON = basal diet without additive; MC = basal diet + 5 mg/kg Microcin C7; OP = basal diet + 40 mg/kg octapeptin. DI: diarrhea index; DR: diarrhea rate. ^a,b^ Means listed in the same row with different superscripts are significantly different (*p* ≤ 0.05).

**Table 4:** The effect of dietary octapeptin supplementation on blood biochemistry in piglets.

Items	CON	MC	OP	SEM	*p*-Value
ALT, U/L	72.83 ^a^	42.58 ^b^	66.67 ^ab^	7.06	0.02
AST, U/L	57.00	73.58	66.67	8.35	0.39
GGT, U/L	53.58	55.50	50.83	3.80	0.69
TBA, μmol/L	42.02	43.99	59.06	9.30	0.39
TP, g/L	49.15	47.84	48.20	0.90	0.58
ALB, g/L	30.77	29.33	29.03	1.22	0.53
GLB, g/L	18.38	18.51	19.11	0.71	0.74
TBil, μmol/L	3.89	3.80	3.53	1.31	0.98
BUN, mmol/L	2.64	2.91	3.09	0.37	0.69
CHO, mmol/L	2.91	2.53	2.68	0.12	0.09
TG, mmol/L	0.74	0.68	0.69	0.06	0.76

ALT: alanine aminotransferase; AST: aspartate aminotransferase; GGT: gamma-glutamyl transferase; TBA: total bile acids; TP: total protein; ALB: albumin; GLB: globulin; TBil: total bilirubin; BUN: blood urea nitrogen; CHO: cholesterol; TG: triglycerides. ^a,b^ Means listed in the same row with different superscripts are significantly different (*p* ≤ 0.05).

**Table 5:** The effect of dietary octapeptin supplementation on serum immunity in piglets.

Items	CON	MC	OP	SEM	*p*-Value
IgG, mg/mL	3.62 ^b^	5.99 ^a^	2.29 ^b^	0.61	<0.01
IgA, mg/mL	0.51 ^b^	0.68 ^a^	0.62 ^ab^	0.05	0.04
IgM, mg/mL	17.74	18.40	15.01	2.02	0.41
IL-2, ng/L	576.16	484.72	305.5	92.17	0.13
IL-6, ng/L	32.60	43.31	24.66	6.70	0.13
IL-10, ng/L	22.27	25.01	15.57	5.62	0.42
TNF-α, ng/L	36.37 ^a^	25.07 ^b^	26.24 ^b^	2.63	0.02

IgG: Immunoglobulin G; IgA: Immunoglobulin A; IgM: Immunoglobulin M; IL-2: Interleukin 2; IL-6: Interleukin 6; IL-10: Interleukin 10; TNF-α: Tumor Necrosis Factor Alpha. ^a,b^ Means listed in the same row with different superscripts are significantly different (*p* ≤ 0.05).

## Data Availability

The data presented in this study are available upon request from the corresponding author.
